# FL-TENB4: A Federated-Learning-Enhanced Tiny EfficientNetB4-Lite Approach for Deepfake Detection in CCTV Environments

**DOI:** 10.3390/s25030788

**Published:** 2025-01-28

**Authors:** Jimin Ha, Abir El Azzaoui, Jong Hyuk Park

**Affiliations:** Department of Computer Science and Engineering, Seoul National University of Science and Technology, Seoul 01811, Republic of Korea; jimin.ha@seoultech.ac.kr (J.H.); abir.el@seoultech.ac.kr (A.E.A.)

**Keywords:** Federated Learning, TinyML, EfficientNetB4, deepfake detection, CCTV environment

## Abstract

The widespread deployment of CCTV systems has significantly enhanced surveillance and public safety across various environments. However, the emergence of deepfake technology poses serious challenges by enabling malicious manipulation of video footage, compromising the reliability of CCTV systems for evidence collection and privacy protection. Existing deepfake detection solutions often suffer from high computational overhead and are unsuitable for real-time deployment on resource-constrained CCTV cameras. This paper proposes FL-TENB4, a Federated-Learning-enhanced Tiny EfficientNetB4-Lite framework for deepfake detection in CCTV environments. The proposed architecture integrates Tiny Machine Learning (TinyML) techniques with EfficientNetB4-Lite, a lightweight convolutional neural network optimized for edge devices, and employs a Federated Learning (FL) approach for collaborative model updates. The TinyML-based local model ensures real-time deepfake detection with minimal latency, while FL enables privacy-preserving training by aggregating model updates without transferring sensitive video data to centralized servers. The effectiveness of the proposed system is validated using the FaceForensics++ dataset under resource-constrained conditions. Experimental results demonstrate that FL-TENB4 achieves high detection accuracy, reduced model size, and low inference latency, making it highly suitable for real-world CCTV environments.

## 1. Introduction

The proliferation of closed-circuit television (CCTV) systems has revolutionized surveillance and security across diverse domains, including urban areas, public transportation, and private businesses. These systems provide continuous monitoring and have become indispensable for crime prevention, evidence collection, and ensuring public safety [1]. In recent years, the integration of advanced technologies has enhanced the capabilities of CCTV, enabling functionalities such as real-time monitoring, facial recognition, and predictive analytics. However, this extensive deployment has also raised concerns about the balance between security benefits and the protection of individual privacy.

CCTV systems inherently involve the collection and processing of vast amounts of personal information. Images and videos captured by these systems often contain sensitive data, such as identifiable facial features, behavior patterns, and other private details of individuals. As a result, safeguarding the privacy and integrity of these data has become critical, particularly in light of evolving cyber threats [2]. Any compromise in the credibility of CCTV data not only violates personal privacy but also undermines the trust and reliability placed in these systems as a source of truth [3].

Amidst these challenges, the emergence of deepfake technology has introduced unprecedented risks to the security of CCTV systems. Deepfakes leverage sophisticated machine learning models, such as generative adversarial networks (GANs), to produce hyper-realistic but fabricated video and audio content [4]. These technologies are no longer confined to academic experimentation but are increasingly accessible and deployable by malicious actors. In the context of CCTV environments, deepfakes can manipulate surveillance footage to obscure evidence, fabricate events, or falsely incriminate individuals, thereby posing significant threats to the authenticity of evidence and the protection of personal information [5].

To mitigate the risks posed by deepfakes, researchers have proposed various AI-based detection solutions aimed at identifying and filtering manipulated content [6]. These solutions leverage advanced machine learning algorithms to detect subtle anomalies in synthetic videos, such as inconsistencies in lighting, facial landmarks, and motion dynamics. For example, tools like STRETCHER utilize deep convolutional neural networks (CNNs) to achieve high detection accuracy [7]. However, despite their effectiveness, existing solutions face critical limitations when applied in real-world CCTV systems. Most state-of-the-art models require extensive computational resources, rendering them unsuitable for resource-constrained environments such as edge devices integrated into CCTV cameras. Additionally, these systems often lack real-time capabilities, making them impractical for dynamic surveillance operations [8].

To address these challenges, this paper proposes a novel lightweight deepfake detection architecture specifically designed for secure CCTV environments. The proposed solution integrates Tiny Machine Learning (TinyML) principles with a Federated Learning (FL) framework, enabling efficient and privacy-preserving real-time video analysis. The architecture employs a TinyCNN based on EfficientNetB4-Lite, a model optimized for edge devices through quantization and scaling techniques. This ensures that the system operates effectively within the computational and memory constraints of CCTV hardware, while the FL approach eliminates the need for centralized data storage, thereby enhancing privacy and scalability.

This paper is structured as follows: the next section provides a comprehensive review of existing deepfake detection solutions and their limitations. The subsequent section details the design and implementation of the proposed TinyCNN-FL-based architecture. A simulation study using the FaceForensics++ dataset evaluates the performance of the proposed system, and the results are analyzed and compared with existing methods. Finally, the paper concludes by summarizing the contributions and discussing potential directions for future research.

## 2. Related Work

Numerous studies have explored the use of surveillance technologies to address challenges such as real-time video processing and deepfake detection in resource-constrained settings. Advances in deep learning and edge computing have enabled significant improvements in the performance of these systems, particularly in terms of accuracy, latency, and computational efficiency. Furthermore, existing research highlights the growing need for robust solutions capable of handling large-scale video streams while maintaining real-time responsiveness. This section critically examines prior work in these areas, identifying their limitations and underscoring the need for an efficient, lightweight, and privacy-preserving approach, such as the one proposed in this paper, for deepfake detection in CCTV video environments.

### 2.1. Existing Studies

The rise of deepfake technology has led to a surge in research dedicated to its detection, yielding a variety of methods and models that aim to identify manipulated visual content effectively. In this section, we review key studies, including those explained in this paper, to provide an understanding of the current landscape of deepfake detection solutions. Each study is described in terms of its techniques, contributions, and limitations, offering insights into the state of the art and areas for improvement. A summary of the explanation is presented in Table 1, and the detailed discussion is as follows.

Chen et al. [9] proposed a framework employing separable self-consistency learning, which incorporates self-supervised techniques to improve the generalization of deepfake detection models across datasets. This method ensures that the model learns consistent patterns from data without requiring extensive labeled samples, thereby addressing the challenge of limited labeled datasets. Although the model demonstrates superior generalization across various manipulation techniques, its reliance on consistency patterns can be vulnerable to highly sophisticated deepfakes that mimic such natural consistencies.

Mallet et al. [10] explored the use of deep learning models, including Xception and MobileNet architectures, for the detection of manipulated video frames. Their study benchmarks these architectures on the FaceForensics++ dataset and highlights the robustness of deep-learning-based models in identifying subtle visual anomalies, such as artifacts in skin textures or irregular motion patterns. Despite achieving high accuracy rates, the resource-intensive nature of these models limits their deployability in edge environments, such as CCTV systems with constrained computational power. Dave et al. [11] investigated the application of hybrid models combining SVM and CNN approaches for deepfake detection. By integrating the CNN’s feature extraction capabilities with the SVM’s classification strength, the hybrid model outperformed traditional methods on standard datasets, achieving high detection accuracy. However, the increased computational complexity of this combined model poses challenges for real-time applications, particularly on low-power devices.

Nguyen et al. [12] presented a deepfake detection approach based on Capsule Networks, designed to model hierarchical spatial relationships in visual data. Capsule Networks excel in capturing spatial inconsistencies introduced by manipulation techniques, such as warping or blurring. While effective in controlled scenarios, this method struggles with large-scale datasets and diverse manipulation types, highlighting the need for further optimization. Rössler et al. [13], the creators of the FaceForensics++ dataset, also provided baseline results using traditional convolutional neural networks (CNNs) for deepfake detection. Their findings demonstrate the effectiveness of CNNs in identifying manipulated regions in high-resolution images. However, the performance of these models declines significantly when dealing with heavily compressed videos, which are common in real-world scenarios. Rana et al. [14] proposed DeepfakeStack, an ensemble-based learning technique that combines multiple deep neural networks to improve deepfake detection performance. By leveraging the strengths of individual models, the ensemble aims to enhance detection accuracy. However, ensemble methods can be computationally intensive, potentially hindering real-time detection capabilities.

Wu et al. [15] introduced an Interactive Two-Stream Network (ITSNet) that explores discriminant inconsistency representations from cross-modal perspectives for deepfake detection. The model integrates both spatial and temporal features to identify manipulations. The complexity of the two-stream architecture may increase computational requirements, affecting deployment in resource-constrained environments. Al-Dulaimi et al. [16] presented a hybrid architecture combining convolutional neural networks (CNNs) and long short-term memory (LSTM) networks for precision deepfake image detection. Utilizing transfer learning, their approach achieved high detection accuracy. However, the hybrid model’s complexity may pose challenges for real-time applications. Kumar et al. [17] proposed a deepfake image detection method using convolutional neural networks (CNNs) combined with transfer learning techniques. The model leverages pre-trained CNN architectures, such as ResNet and EfficientNet, to extract meaningful features from manipulated images. Fine-tuning these pre-trained models on deepfake datasets, the method achieves high detection accuracy while reducing the need for extensive computational resources during training. However, the reliance on pre-trained models can limit the generalizability of the method to novel manipulation techniques not present in the original training datasets.

Bolin Zhang et al. [18] proposed the MVIM network to detect and localize tampered regions in multi-face deepfake videos. The network combines two modules: the Noise Inconsistency Measurement (Noise-IM) module, which identifies noise differences between faces and backgrounds, and the Temporal Inconsistency Measurement (Temporal-IM) module, which captures tampering traces across frames using self-attention and bi-directional convolutions. By fusing features from both modules, MVIM effectively detects manipulated regions and achieves superior performance on benchmark datasets.

Xin Liao et al. [19] proposed the FAMM framework for detecting compressed deepfake videos over social networks. The method introduces a facial-muscle-motion-based approach, leveraging geometric features derived from facial landmarks to represent unnatural facial movements caused by temporal discontinuities in deepfake videos. The framework consists of modules for extracting facial landmarks, constructing facial muscle motion features, and fusing prediction probabilities using Dempster–Shafer theory. By combining difference features with time-series analysis, FAMM decouples compression artifacts from tampering artifacts, achieving superior detection performance compared to state-of-the-art methods on both benchmark and real-world datasets.

Yang Tian et al. [20] proposed an amount-based covert communication scheme over blockchain to address the limitations of existing methods, such as low embedding capacity and high time costs. The scheme introduces the AMASC code, a unique encoding strategy that embeds secret messages into transaction amounts, significantly enhancing embedding capacity. By leveraging Bitcoin’s UTXO structure and efficiently utilizing change addresses, the method minimizes the time cost while maintaining high security and concealment. The approach’s performance, validated on the Bitcoin Testnet, demonstrated eightfold improvement in embedding capacity and superior metrics in security and efficiency compared to state-of-the-art blockchain covert communication methods.

Jiaxin Chen et al. [21] proposed the Signal Noise Separation-based Network (SNIS) for post-processed image forgery detection. The method reformulates the forgery detection problem as a signal noise separation task, where tampered regions are treated as signals and the complex background with post-processing noise as noise. The SNIS framework includes a signal noise separation module to isolate tampered regions, a multi-scale feature learning module using parallel atrous convolution to extract global features, and a feature fusion module for enhancing boundary details. Extensive experiments show that SNIS significantly outperforms state-of-the-art methods in robustness against post-processing attacks and in cross-dataset evaluations.

Abbas Yazdinejad et al. [22] proposed a robust privacy-preserving Federated Learning model to counteract model poisoning attacks while maintaining accuracy and efficiency. The approach incorporates an internal auditor that analyzes encrypted gradients using Gaussian Mixture Models (GMMs) and Mahalanobis Distance (MD) to differentiate between benign and malicious updates. By employing Additive Homomorphic Encryption (AHE), the model ensures data confidentiality without significant computational or communication overhead. Experimental results demonstrate superior accuracy and resilience against both targeted and untargeted attacks in IID and non-IID settings compared to existing methods.

Danyal Namakshenas et al. [23] proposed a Federated Quantum-Based Privacy-Preserving Threat Detection Model to address security and privacy challenges in the Consumer Internet of Things (CIoT). The model integrates Federated Learning (FL) with quantum-based registration and authentication mechanisms to validate client integrity and employs Additive Homomorphic Encryption (AHE) to secure data privacy. These advancements ensure robust threat detection while safeguarding sensitive data from malicious actors and quantum computing threats. Experimental results on the Edge-IIoTset and N-BaIoT datasets demonstrated superior performance, achieving average accuracies of 94.93% and 91.93%, respectively, highlighting the model’s efficiency in diverse CIoT environments.

Danyal Namakshenas et al. [24] proposed the IP2FL model, an Interpretation-Based Privacy-Preserving Federated Learning framework tailored for Industrial Cyber–Physical Systems (ICPSs). This model integrates Additive Homomorphic Encryption (AHE) for secure aggregation of client data, ensuring privacy while reducing computational overhead. Additionally, it employs Shapley Values (SVs) for enhanced explainability, providing insights into model decisions and feature importance. Dual feature selection methods further optimize performance by efficiently managing high-dimensional data. Experimental results using Edge-IIoTset and N-baIoT datasets validate the model’s scalability, robust privacy measures, and high detection accuracy in complex ICPS environments.

Abbas Yazdinejad et al. [25] proposed the Block Hunter framework, a Federated Learning (FL)-based threat detection model for blockchain-based IIoT networks. The framework employs a cluster-based architecture for anomaly detection, integrating machine learning models such as Neural Encoder–Decoder (NED), Isolation Forest (IF), and Cluster-Based Local Outlier Factor (CBLOF) for enhanced efficiency and accuracy. Block Hunter leverages FL to ensure data privacy by training models locally while sharing only model updates. Evaluation on IIoT-related datasets demonstrated its superior anomaly detection performance, achieving high accuracy, precision, and recall while reducing bandwidth and computational overhead.

Bolin Zhang et al. [26] proposed the Multi-View Inconsistency Measurement (MVIM) network to improve deepfake detection and localization in multi-face scenarios. The MVIM network integrates the Noise Inconsistency Measurement (Noise-IM) module, which identifies tampered regions by analyzing inconsistent noise patterns between real and manipulated faces, and the Temporal Inconsistency Measurement (Temporal-IM) module, which detects abrupt changes across frames using self-attention and bi-directional convolutions. These modules are combined via a feature fusion module (FFM) to aggregate multi-scale features for more accurate detection and localization. Experiments demonstrate that MVIM significantly outperforms state-of-the-art methods on benchmark datasets, excelling in both classification and localization accuracy.

### 2.2. Key Considerations

The development of a robust deepfake detection system for CCTV environments necessitates a careful consideration of several critical factors, including efficiency, real-time capabilities, security and privacy, resource constraints, and scalability. Addressing these factors ensures that the proposed architecture can meet the demands of modern surveillance systems while overcoming the limitations of existing solutions.

1. Efficiency: Efficiency is a paramount concern in designing a deepfake detection system, particularly for CCTV environments where high volumes of video data require analysis. Conventional deep learning models often prioritize accuracy at the expense of computational feasibility, making them unsuitable for resource-limited edge devices. By leveraging EfficientNetB4-Lite, a lightweight version of EfficientNetB4 specifically optimized for high-definition video deepfake detection, the system achieves a significant balance between accuracy and computational efficiency. The incorporation of compound scaling techniques and further optimization through Tiny Machine Learning (TinyML) enable the system to maintain high detection accuracy while significantly reducing model size and computational overhead. These refinements ensure superior detection performance in resource-constrained environments, making EfficientNetB4-Lite an ideal solution for deployment on edge devices without compromising performance [27].
2. Real-time: Another essential consideration is the ability to process video data in real-time. CCTV systems require immediate analysis of incoming video streams to detect manipulations as they occur. The proposed solution leverages the EfficientNetB4-Lite model, an optimized CNN architecture that achieves both computational efficiency and rapid inference speed. The lightweight nature of EfficientNetB4-Lite, combined with TinyML optimizations such as quantization and pruning, ensures that the detection system can handle real-time processing demands. This capability is critical for maintaining the integrity of surveillance footage and enabling prompt responses to potential security threats [28].
3. Security and Privacy: The protection of security and privacy is equally vital in the context of CCTV systems. Traditional deep learning approaches often rely on centralized data processing, which poses significant risks to data privacy and security. By adopting a Federated Learning (FL) framework, the proposed architecture addresses these concerns effectively. Federated Learning enables decentralized model training, where sensitive data remain on local devices, and only model updates are shared with a central server. This approach not only preserves user privacy but also reduces the risks associated with data breaches and unauthorized access, aligning with the principles of modern data protection regulations [29].
4. Resource constraints: Resource constraints are a significant challenge, as CCTV systems typically operate on edge devices with limited processing power and memory capacity. To address this issue, the design of the proposed solution incorporates TinyML optimizations to ensure compatibility with such hardware limitations. Specifically, quantization techniques such as dynamic range quantization and full integer quantization are applied to EfficientNetB4-Lite. These optimizations significantly reduce model size and inference latency while maintaining detection accuracy. As a result, the system becomes highly feasible for deployment in real-world CCTV environments, even on devices with minimal computational resources [30].
5. Scalability: The scalability is a critical consideration for the deployment of deepfake detection systems in large-scale surveillance networks. The Federated Learning framework not only supports decentralized training but also ensures scalability by allowing multiple devices to collaboratively train a global model. This approach eliminates the need for extensive infrastructure and centralized computation, making it highly adaptable to diverse and distributed CCTV systems [31].

In summary, the proposed architecture addresses key considerations that are essential for effective deepfake detection in CCTV environments. By ensuring efficiency, real-time processing, security and privacy, resource compatibility, and scalability, the design is tailored to meet the practical demands of modern surveillance systems. Through the application of TinyML and the further optimization of EfficientNetB4-Lite, this system achieves exceptional detection accuracy and operational efficiency even in resource-limited environments.

## 3. Proposed Architecture

The proposed TinyFL-CNN-based XAI architecture is designed to detect and mitigate network anomalies in CCTV environments through a lightweight Federated Learning architecture combined with a convolutional neural network. The system model comprises key components that collaboratively work to efficiently identify and mitigate abnormal states in the network.

### 3.1. System Overview

**Figure 1:**
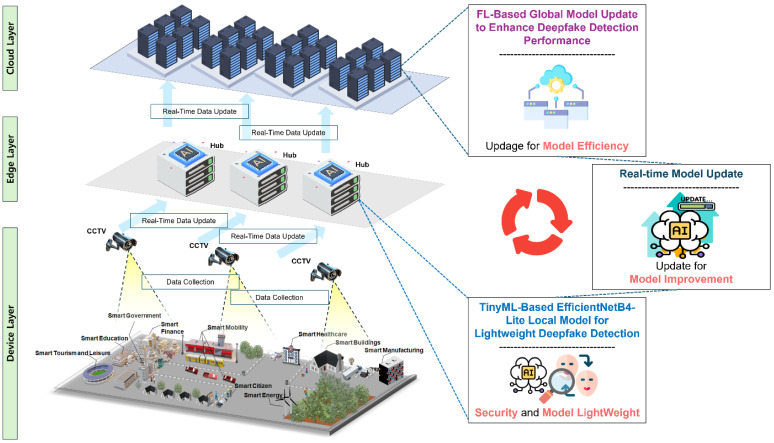
Architecture overview.

The proposed system introduces a lightweight yet robust architecture for detecting deepfakes in video streams, specifically designed for deployment in resource-constrained CCTV environments. The system integrates Tiny Machine Learning (TinyML) principles with a Federated Learning (FL) framework to achieve a balance between computational efficiency, scalability, and privacy preservation. By leveraging the optimized EfficientNetB4-Lite model, the architecture ensures real-time processing capabilities while maintaining high detection accuracy, even under the limited hardware constraints of edge devices. This paper proposes a Federated-Learning-enhanced Tiny EfficientNetB4-Lite approach for deepfake detection (FL-TENB4) to efficiently, and in real time, detect deepfakes in CCTV video streams under constrained environments. Our proposed architecture can be divided into three layers: a (1) device layer, (2) edge layer, and (3) cloud layer. An overview of the architecture is depicted in Figure 1 bellow.

Device Layer: The device layer serves as the foundation of the architecture by facilitating data collection through CCTV systems deployed across various environments. These environments can range from public spaces such as transportation hubs, shopping centers, and streets to private spaces like offices and residential complexes. CCTV cameras in these settings capture continuous video streams, often under diverse conditions, including varying lighting, motion patterns, and environmental noise. The device layer ensures that raw video data are gathered efficiently and transmitted securely to the subsequent layer for processing. This adaptability across heterogeneous conditions allows the system to function robustly regardless of external challenges, ensuring that critical video data are captured reliably.

Edge Layer: The edge layer is the heart of the system’s detection capabilities, employing a TinyEfficientNetB4-Lite-based deepfake detection algorithm as its local model. TinyEfficientNetB4-Lite, a highly optimized and lightweight variant of the EfficientNet family, is specifically tailored for detecting deepfakes in high-definition video. Through Tiny Machine Learning (TinyML) optimizations such as quantization and pruning, the model is capable of performing accurate and efficient inference on edge devices with limited computational resources. This layer processes video frames received from the device layer in real time, analyzing them for subtle inconsistencies that are characteristic of deepfakes, such as irregularities in lighting, motion artifacts, or unnatural facial expressions. By operating locally on edge devices, the edge layer ensures timely detection without the need for centralized resources, significantly reducing latency and ensuring privacy compliance by keeping raw data on the devices.

Cloud Layer: The cloud layer completes the architecture by managing global model updates using a Federated Averaging (FedAvg) framework. Federated Learning allows local models trained on individual edge devices to contribute to a global model without transferring sensitive video data to a central server. Instead, each device sends encrypted updates of its local model to the cloud layer, where the FedAvg algorithm aggregates these updates to refine the global model. This decentralized approach not only enhances data privacy but also ensures scalability by enabling collaborative training across a distributed network of devices. The iterative refinement of the global model ensures that it evolves continuously, improving its robustness and adaptability to emerging deepfake generation techniques.

The proposed architecture adopts a distributed structure, where Section 3.2 and Section 3.3 focus on the local model situated at the edge layer and the global model located at the cloud layer, respectively.

### 3.2. TENB4: TinyML-Based EfficientNetB4-Lite Local Model for Deepfake Detection

The proposed architecture employs a TinyML-based EfficientNetB4-Lite local model to detect deepfakes (TENB4) with high accuracy while addressing the constraints of resource-limited environments. This local model is optimized for deployment on edge devices, leveraging quantization and other TinyML techniques to ensure real-time performance without sacrificing detection accuracy. Its modular design and efficient algorithmic framework enable effective video analysis, contributing significantly to the overall robustness of the system. The TENB4 model for deepfake detection is composed of two key components: (1) an EfficientNetB4-Lite-based deepfake detection model designed for high accuracy and (2) model compression through TinyML optimization to enhance efficiency.

#### 3.2.1. EfficientNetB4-Lite-Based Deepfake Detection Model

EfficientNetB4-Lite is a lightweight adaptation of the EfficientNet family of convolutional neural networks (CNNs), specifically designed for resource-constrained environments [32]. Through compound scaling, EfficientNetB4-Lite adjusts the network’s depth, width, and resolution in a balanced manner to achieve optimal performance. The lightweight nature of this model makes it particularly suited for edge-based applications such as CCTV surveillance, where computational resources are limited. By employing quantization and pruning, the model achieves a substantial reduction in size and computational overhead, enabling real-time inference while maintaining high accuracy. These characteristics make EfficientNetB4-Lite an ideal choice for detecting subtle inconsistencies in deepfake videos, such as lighting irregularities, facial distortions, and motion artifacts.

Algorithm 1 focuses on the real-time inference capabilities of the EfficientNetB4-Lite model within the local environment. The process begins with initializing a TinyML inference engine on an edge device, preparing it to load the pre-trained EfficientNetB4-Lite model optimized with quantization techniques. The algorithm operates continuously, capturing video frames in real time from CCTV streams. Each frame undergoes preprocessing, which includes resizing to 260 × 260 pixels and normalizing pixel values to ensure compatibility with the model. The preprocessed frame is then converted into a tensor format, enabling efficient input to the detection model.

Once the model processes the frame, it outputs probabilities indicating whether the frame is real or fake. If the probability of a fake frame exceeds a predefined detection threshold, the system triggers an alert to notify the relevant monitoring authority. This alert mechanism ensures that manipulations in video streams are flagged promptly, maintaining the integrity of surveillance operations. The detailed operation process is explained in Algorithm 1.
**Algorithm 1:** EfficientNetB4-Lite based Deepfake Detection Model1: **Input:** Real-time image frame stream from CCTV device ($Frame_{t}$), Pre-trained EfficientNetB4-Lite model optimized for TinyML (Local_Model) 2: **Output:** Detection result (Real or Fake) for each frame. 3: **Process:**
4: Initialize TinyML inference engine on Edge Device 5: Load Local_Model (EfficientNetB4-Lite optimized with quantization) 6: **while** True do 7:   $Frame_{t}$ = Capture next frame from CCTV stream 8:   $Preprocess_{Frame}=Resize\left(Frame_{t},\left(260,260\right)\right),\;Normalize(Frame_{t})$
9:   Model_Input=Convert_to_Tensor(Preprocess_Frame)
10:   Model_Output=Local_Model_Inference(Model_Input)
11:   **if** Model_Output[Fake_Probability]>Detection_Threshold **then** 12:     Result=“Fake” 13:     Trigger_Alert(System) 14:   **else**
15:     Result=“Real” 16:   **end if** 17: **end while**

1. Input and Initialization: The algorithm takes real-time image frames Frame_t, captured sequentially from a CCTV device’s video stream, as an input, and a pre-trained EfficientNetB4-Lite model optimized for TinyML deployment. The algorithm initializes the TinyML inference engine on the edge device. This engine is implemented using a TensorFlow Lite (TFLite) 2.17.0 interpreter, which facilitates the execution of the EfficientNetB4-Lite model optimized with quantization techniques (such as dynamic range or full integer quantization).
  Real-Time Frame Acquisition: The algorithm continuously acquires video frames from the CCTV stream in a loop. Each frame $Frame_{t}$ represents a snapshot in time and is a three-dimensional tensor $Frame_{t}$ ∈ R^(H×W×3)^, where H and W are the original dimensions of the image. These frames are processed sequentially to maintain real-time performance.
2. Preprocessing: Each frame undergoes a series of preprocessing steps to prepare it for input into the EfficientNetB4-Lite model.
  - Resizing: The original frame $Frame_{t}$ is resized to 260 × 260 pixels, the input dimension expected by the model. This resizing ensures uniformity and compatibility while maintaining critical visual details for detection. Mathematically:
    (1) $$Preprocess_{Frame}=Resize\left(Frame_{t},\left(260,260\right)\right)$$
  - Normalization: The pixel values of the resized frame are normalized to the range [0, 1] by dividing each pixel intensity by 255. This normalization stabilizes the model’s numerical computations during inference. Mathematically:
    (2) $$Preprocess\_Frame=\frac{Preprocess_{Frame}}{255.0}$$
  - Tensor Conversion: The normalized frame is converted into a tensor format, with an additional batch dimension added for compatibility with the model. The resulting tensor has a shape of 1×260×260×3.
3. Model Inference: The preprocessed frame is passed to the EfficientNetB4-Lite model for inference. This model, which has been optimized for TinyML using quantization, predicts probabilities for two outcomes:
  - $P_{Real}$: The probability that the frame is authentic.
  - $P_{Fake}$: The probability that the frame is a deepfake.
  - The output satisfies the condition $P_{Real}+P_{Fake}=1$. The detection output is represented as follows:
    (3) $$Model_{Output}=\left[P_{Real},P_{Fake}\right]$$
4. Decision-Making and Alert Generation: The algorithm applies a predefined detection threshold $T_{Detection}$ to the fake probability $P_{Fake}$ to classify the frame:
  - If $P_{Fake}>T_{Detection}$, the frame is classified as fake, and the system triggers an alert to notify the monitoring authority.
  - Otherwise, the frame is classified as real, and no further action is taken.

This decision-making step is critical for real-time operation, ensuring that only frames likely to be manipulated are flagged for further investigation.

5. Continuous Processing: The algorithm operates in an infinite loop, repeatedly capturing, processing, and analyzing frames from the video stream. This ensures uninterrupted surveillance and prompt detection of deepfakes in real time.

#### 3.2.2. Model Compression Through TinyML Optimization

Tiny Machine Learning (TinyML) refers to the deployment of machine learning models on resource-constrained edge devices, such as microcontrollers and embedded systems, to perform inference locally. By utilizing techniques like model quantization, pruning, and knowledge distillation, TinyML ensures that even complex deep learning algorithms can run efficiently with minimal computational power, memory, and energy consumption. Unlike traditional ML, which often relies on centralized cloud-based systems, TinyML processes data locally, reducing latency and ensuring privacy by keeping sensitive data on the device. Its ability to execute models in real time, with power consumption typically in the milliwatt range, makes TinyML ideal for low-power IoT devices and applications like surveillance, healthcare, and manufacturing.

In the context of deepfake detection, TinyML offers several key advantages. By enabling localized inference, it allows real-time analysis of video streams directly on edge devices, such as CCTV cameras, eliminating the need for high-bandwidth data transmission to centralized servers. TinyML optimizations, such as quantization, significantly reduce the size and computational overhead of models like EfficientNetB4-Lite while maintaining high detection accuracy. This makes it feasible to deploy effective deepfake detection systems in resource-limited environments, such as battery-operated or low-power surveillance networks. Additionally, TinyML’s scalability and energy efficiency allow widespread deployment across distributed systems, ensuring cost-effective and privacy-preserving solutions for modern surveillance challenges.

Algorithm 2 systematically outlines the optimization process for deploying the EfficientNetB4-Lite model on resource-constrained edge devices using TinyML quantization techniques. The goal of this process is to significantly reduce the model’s size, computational complexity, and memory requirements while preserving the detection accuracy required for reliable deepfake detection. This detailed explanation expands on the steps described in the algorithm, emphasizing their technical aspects and practical implications. The detailed operation process of Algorithm 2 is explained as follows:
**Algorithm 2:** Quantization (TinyML Optimization)1: **Input:** Pre-trained Local_Model (EfficientNetB4-Lite),       Quantization type (Dynamic Range, Full Integer, or Float16) 2: **Output:** Optimized TFLite Model (TFLite_Model) 3: **Process:**
4:   Export the Local_Model from training environment 5:   Convert Local_Model to TensorFlow Lite format: 6:         TFLite_Model=TFLiteConverter(Local_Model) 7:   Apply Quantization techniques: 8:         a. Dynamic Range Quantization: 9:           Quantized Weights:                $W_{INT8}=Round(\frac{WFP32}{Scale})+Zero\_Point$ 10:         b. Full Integer Quantization: 11:           Quantized Activations:                   $A_{INT8}=Quantize(A_{FP32})$ 12:         c. Float16 Quantization: 13:           Quantized Weights: 
                 $W_{FP16}=Cast(W_{FP32}\to FP\_16)$ 14:  Deploy the Quantized TFLite Model to Edge Device

Exporting the Pre-Trained Model: The process begins by exporting the pre-trained EfficientNetB4-Lite model from the training environment. This model has been previously trained on a dataset optimized for deepfake detection to ensure that it captures the subtle artifacts and patterns associated with manipulated video frames. The exported model serves as the foundation for the quantization process. Exporting involves saving the model in a format compatible with TensorFlow’s tools, ensuring that it retains all trained parameters such as weights, biases, and architectural configurations. This exported model, while accurate, is typically in a 32-bit floating-point (FP32) representation, which is computationally expensive for edge devices.

Conversion to TensorFlow Lite (TFLite) Format: The exported FP32 model is then converted into TensorFlow Lite (TFLite) format using the TensorFlow Lite Converter. This step is critical for adapting the model to the requirements of edge device environments, where lightweight inference engines like TFLite interpreters are employed. The conversion involves restructuring the model to make it compatible with hardware and software constraints while retaining the original architecture. During this step, the model’s compatibility with post-training optimizations, such as quantization, is established. The TFLite format supports reduced-precision computations, which are necessary for deploying a model in environments where power efficiency, memory, and processing constraints are significant considerations.

Quantization Techniques: The core of Algorithm 2 lies in applying quantization techniques to the TFLite model. These techniques reduce the numerical precision of the model’s weights and activations, achieving significant memory savings and faster inference speeds. The following methods are employed during this step, as summarized in Table 2:

- Dynamic Range Quantization: Dynamic range quantization compresses the model weights by converting them from FP32 to 8-bit integer (INT8) format, while keeping the activations in FP32. This approach does not require calibration data and is relatively straightforward to implement. It offers a balance between model size reduction and preservation of detection accuracy, although it provides a lower level of compression compared to other techniques. This quantization method is particularly suitable for environments where simplicity and rapid deployment are essential, such as general-purpose CPUs or older edge devices without native INT8 support. While dynamic range quantization achieves moderate compression, its use of FP32 activations limits latency reduction. The quantization process is represented mathematically as
  (4) $$W_{INT8}=Round(\frac{W_{FP32}}{Scale})+Zero\_Point$$
  where $W_{INT8}$ is the quantized weight, $W_{FP32}$ is the original weight, and Scale and Zero_Point are calibration factors determined during the conversion process.
- Full Integer Quantization: Full integer quantization compresses both the weights and activations to INT8 format. This method requires calibration data to ensure that the quantized model retains detection accuracy close to the original FP32 model. By quantizing the entire inference pipeline, including activations, full integer quantization maximizes latency reduction and power efficiency. It is thus well-suited for real-time applications on resource-constrained edge devices. This technique is ideal for scenarios where low latency and power efficiency are critical, such as real-time deepfake detection on devices like Raspberry Pi 4 or other ARM-based processors with native INT8 support. The calibration data used during this process ensure the quantized model approximates the behavior of the FP32 model. The quantization process is mathematically expressed as
  (5) $$A_{INT8}=Quantize\left(A_{FP32}\right)$$
  where $A_{INT8}$ and $A_{FP32}$ represent the quantized and original activations, respectively.
- Float16 Quantization: Float16 (FP16) quantization reduces model weights to 16-bit floating-point precision. Compared to integer quantization methods, Float16 achieves a moderate level of compression while maintaining higher numerical precision. This method offers a balance between reducing memory usage and preserving accuracy, making it ideal for edge devices with slightly higher computational capabilities. This approach is most effective in deployments requiring higher numerical precision, such as edge devices with GPUs or specialized hardware like NVIDIA Jetson, which natively support FP16 computations. While it provides limited latency reduction compared to full integer quantization, it ensures minimal degradation in model accuracy. The mathematical transformation for Float16 quantization is as follows:
  (6) $$W_{FP16}=Cast(W_{FP32}\to FP\_16)$$
  where $W_{FP16}$ represents the compressed weight, and $W_{FP32}$ is the original weight.

**Table 2:** Computational trade-offs of quantization techniques.

Quantization Type	Compression	Accuracy Preservation	Latency Reduction	Calibration Data	Use Case
Dynamic Range Quantization	Moderate	High	Low	Not Required	Prototyping, general-purpose CPUs
Full Integer Quantization	High	Moderate	High	Required	Real-time detection on low-power devices
Float16 Quantization	Moderate	Very High	Moderate	Not Required	GPUs or FP16-supported hardware

Deployment of the Optimized Model: Once the quantization process is complete, the resulting quantized TFLite model is deployed to the edge device. This step involves transferring the model to the device’s storage and initializing the TinyML inference engine, which will execute the model for real-time deepfake detection. The optimized model is now ready for efficient inference, capable of analyzing incoming frames from the CCTV system with reduced computational and memory demands. The quantized model demonstrates significant improvements in performance metrics, including reduced inference latency, decreased memory consumption, and lower power requirements. These optimizations make the deployment viable on devices with minimal hardware resources, such as embedded processors in CCTV systems.

The TinyML-Based EfficientNetB4-Lite Local Model (TENB4) is designed to achieve high accuracy in deepfake detection while operating on resource-constrained edge devices. This lightweight model leverages TinyML techniques such as quantization and pruning to minimize computational complexity, enabling real-time video analysis on devices like CCTV systems. TENB4 integrates modular components, including an optimized detection model and efficient algorithms for preprocessing and inference, to provide robust performance in detecting manipulations in high-definition video streams.

### 3.3. FL-Based Global and Local Model Update to Enhance Deepfake Detection Performance

The proposed architecture incorporates a Federated Learning (FL) model to enable collaborative model training across distributed edge devices without sharing sensitive data. This design enhances the deepfake detection system’s performance while preserving privacy. The FL model updates the global model using Federated Averaging (FedAvg) and synchronizes the local models on edge devices with the updated global model. This dual-layered update mechanism ensures the system remains adaptive to emerging deepfake manipulation techniques while maintaining computational efficiency.

Algorithm 3 outlines the Federated Averaging (FedAvg) process, a cornerstone of the Federated Learning (FL) framework. This algorithm enables collaborative training across distributed edge devices, ensuring that a global model benefits from diverse local datasets without transmitting sensitive data. The detailed steps of the algorithm are as follows:
**Algorithm 3:** Global Model Update with Federated Averaging (FedAvg)1: **Input:** Local_Model_Updates_i from N Edge devices (i = 1 to N), Initial Global_Model parameters ($w_{0}$) 2: **Output:** Updated Global_Model parameters ($w_{t+1}$) 3: **Process:**
4:  Initialize Global_Model with parameters $w_{0}$. 5:  **for** each Federated Learning round t = 1 to T do 6:    Collect Local_Model_Updates_i from all Edge devices i = 1 to N. 7:    Perform Federated Averaging: 8:      $w_{t+1}\leftarrow \;\frac{1}{N}*\sum_{i=1}^{N}w_{i}^{\left(t\right)}\;$
9:    Update Global_Model parameters $w_{t+1}$ 10:     Distribute Global_Model parameters $w_{t+1}$ to all Edge devices. 11:  **end for**
12:  **Return** final Global Model

1. Initialization of the Global Model: The first step in the Federated Averaging process is to initialize the global model with the initial parameters $w_{0}$ These initial parameters can either be pre-trained model weights or randomly initialized values. This global model serves as the starting point for the training and aggregation process across multiple edge devices. The central server (aggregator) stores the global model and coordinates the update process.

Federated Learning Rounds: Federated Learning proceeds in multiple rounds. In each round, local models on edge devices are trained using their respective datasets, and the global model is updated based on the aggregated local updates. Each round consists of several steps:

- Local training: Each edge device trains its local model on its own dataset.
- Local updates: After training, the local device sends its model updates (parameters) to the central server.
- Global model update: The server aggregates the local updates and computes the new global model using Federated Averaging.
- Global model distribution: The updated global model is sent back to all edge devices for further local training in the next round.

Collect Local Model Updates from Edge Devices**:** Once the global model is initialized and the first round of local training is completed on all edge devices, the next step is to collect the local model updates from all participating devices. Each device sends its updated model parameters $w_{i}^{\left(t\right)}$ (where t denotes the current round of training) to the central server. These updates contain the learned parameters from local data and are used to refine the global model.

2. Perform Federated Averaging: After collecting all the local updates from the devices, the central server performs Federated Averaging. In this step, the server computes a weighted average of the local model parameters based on the size of the local datasets on each device. The updated global model $w_{t+1}$ is computed as follows:
  (7) $$w_{t+1}\leftarrow \;\frac{1}{N}*\sum_{i=1}^{N}w_{i}^{\left(t\right)}\;$$
  where N is the total number of participating edge devices and $w_{i}^{\left(t\right)}$ are the parameters of the local model after training on device i. This step ensures that each device’s contribution to the global model is proportional to the amount of data it has, which helps preserve the performance of the global model across a distributed network.

Update the Global Model: Once the global model $w_{t+1}$ is computed, the server updates the global model with the new aggregated parameters. This new global model incorporates the knowledge gained from all the participating edge devices, making it more robust and generalized.

Distribute Updated Global Model: After updating the global model, the server distributes the new model parameters $w_{t+1}$ to all participating edge devices. This allows each edge device to begin training its local model using the updated global model as a starting point for the next round of local training.

Repeat Until Convergence: Steps 3 to 6 are repeated for several Federated Learning rounds. In each round, local models are further trained, aggregated, and refined to create an increasingly accurate global model. The process continues until a predefined number of rounds T is reached or until the performance of the global model stabilizes, indicating that further updates will yield diminishing returns.

Return the Final Global Model: At the end of the Federated Learning process, after completing all the rounds, the final global model $w_{T}$ is returned. This model reflects the collective learning of all participating edge devices, making it more robust for deepfake detection across diverse environments.

In conclusion, this paper proposes the FL-TENB4 architecture, a Federated-Learning-enhanced Tiny EfficientNetB4-Lite approach, to efficiently detect deepfakes in CCTV video streams under constrained environments. This approach leverages the privacy-preserving and adaptive capabilities of Federated Learning to address the challenges posed by real-time deepfake detection in distributed systems.

## 4. Simulation and Analysis of the Proposed Architecture

### 4.1. Simulation

The simulations are conducted to rigorously evaluate the performance of the proposed architecture under various conditions. Specifically, the experiments are designed to test the system’s scalability, robustness, and efficiency across different scenarios, ensuring its applicability to real-world use cases. Key performance metrics such as response time and accuracy are measured and analyzed. Comparative simulations with existing architectures are also included to highlight the advantages of the proposed approach.

#### 4.1.1. Simulation Environment

The simulation environment for the proposed FL-TENB4 architecture was designed to emulate the constraints of resource-limited edge computing in CCTV networks. In this study, experiments were conducted using data collected from our own CCTV devices alongside the FaceForensics++ (FF++) dataset. However, the CCTV data collected in-house had limitations, including low resolution, a limited number of samples, and the absence of diverse deepfake generation techniques. Therefore, the FF++ dataset was used in parallel due to its well-documented advantages. FF++ is a widely adopted benchmark dataset in deepfake detection research, providing diverse deepfake generation methods and various compression levels (raw, lightly compressed, and heavily compressed). By combining these datasets, the proposed model’s performance could be validated more comprehensively across different scenarios.

The FaceForensics++ dataset, featuring diverse real and manipulated video frames across multiple deepfake techniques and compression levels, was split into training, validation, and test sets (70:15:15). Hardware included Raspberry Pi 4 Model B boards (4 GB RAM, Quad-core Cortex-A72 CPU) at the edge layer for real-time inference and an NVIDIA Tesla V100 GPU (32 GB VRAM) in the cloud layer for global model aggregation. TensorFlow Lite optimized the Tiny EfficientNetB4-Lite model for edge deployment through quantization and pruning, while TensorFlow Federated 2.17.0. managed privacy-preserving training with encrypted updates.

Preprocessing involved resizing video frames to 260 × 260 pixels and normalizing them to simulate real-time CCTV feeds, accommodating environmental variability. Tools like PowerStat and TensorFlow Lite benchmarks measured energy consumption, latency, and memory usage, validating the architecture’s ability to achieve accurate deepfake detection with efficiency and data privacy. The simulation environment is summarized in Table 3. This environment effectively replicated real-world conditions to demonstrate FL-TENB4’s scalability and robustness in distributed surveillance systems.

#### 4.1.2. Simulation Result

The simulation results demonstrate the efficiency and robustness of the proposed FL-TENB4 model for deepfake detection in resource-constrained environments. Using the FaceForensics++ dataset, which encompasses a diverse range of manipulated and real video frames, the model achieved outstanding performance across key metrics, validating its suitability for real-world deployment.

**Figure 2:**
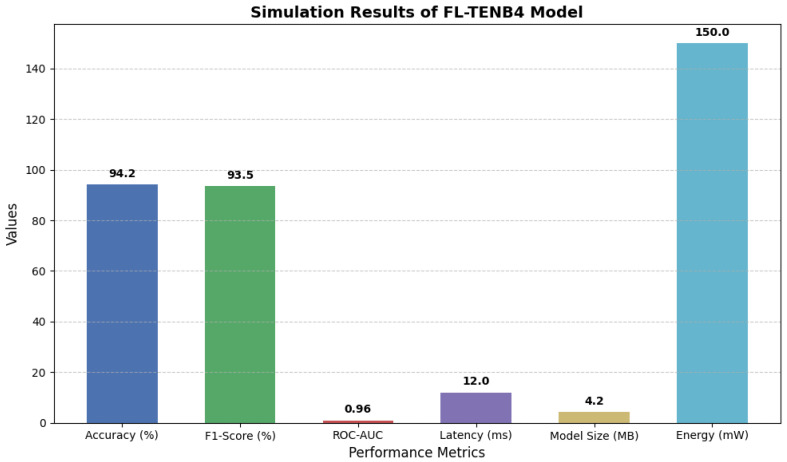
Simulation results of FL-TENB4 model.

As illustrated in Figure 2, the FL-TENB4 model delivered a detection accuracy of 94.2% and an F1-Score of 93.5%, highlighting its ability to reliably distinguish between authentic and fake frames. Additionally, the model achieved an ROC-AUC score of 0.96, reflecting its excellent discriminatory capability. These results were achieved with a low inference latency of 12 ms per frame, demonstrating real-time processing capabilities suitable for edge-based surveillance systems.

The FL-TENB4 model, optimized using quantization and pruning techniques, achieved a compact size of 4.2 MB, enabling its deployment on low-resource edge devices. Furthermore, the energy consumption was measured at 150 mW during inference, underscoring its energy efficiency and compatibility with IoT and edge hardware. The model’s lightweight nature and optimization make it an ideal solution for distributed surveillance networks that require privacy-preserving, real-time deepfake detection.

The following graph illustrates the key simulation results, including accuracy, latency, model size, and energy consumption, providing a visual comparison of the model’s performance.

### 4.2. Analysis

The performance of the proposed FL-TENB4 architecture was evaluated through a rigorous set of simulations using the FaceForensics++ dataset, a well-established benchmark for deepfake detection. The experiments focused on quantifying the effectiveness of the architecture under both constrained resource environments and varying levels of data compression (C0, C23, and C40). The analysis compared FL-TENB4 against several state-of-the-art approaches, including Multi-View Inconsistency Detection, Self-Consistency Learning, XceptionNet, and CNN-SVM-based models, using both performance and efficiency metrics. The performance of FL-TENB4 across different compression levels is summarized in Table 4, and the detailed explanation is as follows.

The FL-TENB4 model was evaluated across three versions of the FaceForensics++ dataset—C0 (raw), C23 (lightly compressed), and C40 (heavily compressed)—to assess its robustness under varying compression levels. C0 represents uncompressed, high-quality frames with detailed visual information, while C23 includes standard compression artifacts, simulating typical video quality. C40 contains severe compression artifacts, mimicking low-quality CCTV footage. The model achieved the highest performance on the C0 dataset with an accuracy of 96.5%, demonstrating its ability to detect manipulations in high-quality videos. On the C23 dataset, which most closely resembles real-world CCTV conditions, the model maintained robust performance with an accuracy of 94.2%. For the C40 dataset, accuracy decreased to 89.8% due to the significant visual detail loss caused by heavy compression. Given the practical relevance of C23 to real CCTV environments, further comparative analyses were conducted using this dataset to validate the model’s applicability to realistic surveillance scenarios.

**Figure 3:**
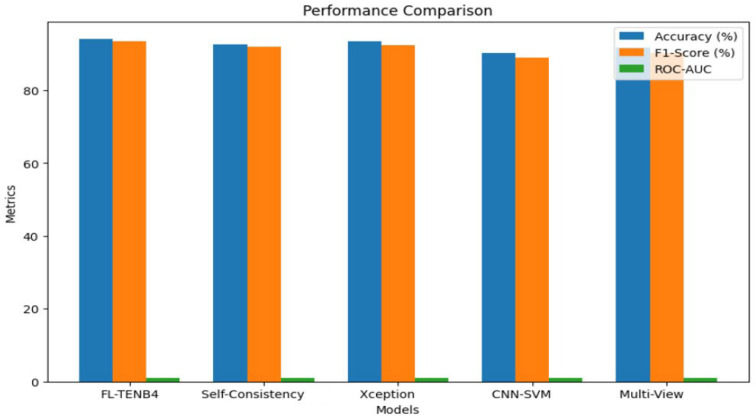
Performance comparison.

**Figure 4:**
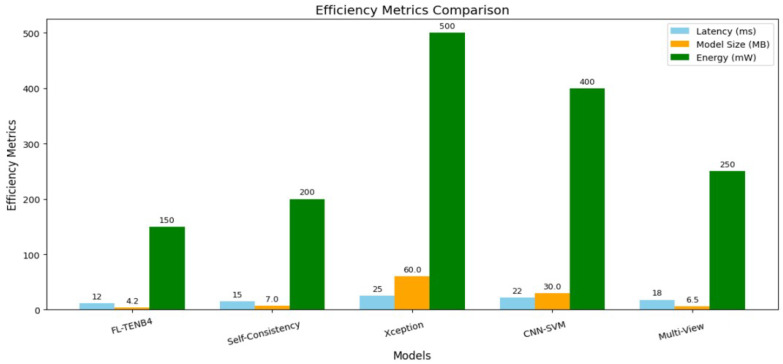
Comparison of efficiency metrics.

As illustrated in Figure 3, Figure 4, and Table 5, the proposed FL-TENB4 architecture is compared against four existing approaches, highlighting its performance, efficiency, bandwidth utilization, and latency variability. The comparison evaluates FL-TENB4’s capabilities alongside state-of-the-art models such as Multi-View Inconsistency, CNN-SVM, XceptionNet, and Self-Consistency Learning. These analyses underscore the distinct advantages of FL-TENB4 in delivering high detection accuracy, reduced latency, optimized bandwidth utilization, and scalability for deployment in resource-constrained CCTV environments.

Performance: In terms of performance metrics, the FL-TENB4 architecture demonstrated a high level of accuracy, achieving 94.2% in the detection of manipulated video frames. This result outperformed several other approaches, including Multi-View Inconsistency (91.8%) and CNN-SVM (90.2%), while showing comparable performance to XceptionNet (93.5%). Similarly, FL-TENB4 achieved a robust F1-Score of 93.5%, reflecting its balanced performance in precision and recall. The ROC-AUC value of 0.96 further validated the system’s ability to discriminate between authentic and deepfake frames, indicating strong generalization across diverse conditions and manipulation techniques. These results emphasize the effectiveness of leveraging TinyML optimizations, which enhance the detection capabilities of the EfficientNetB4-Lite model even under stringent hardware limitations.

Efficiency: The efficiency metrics further underscored the viability of FL-TENB4 for real-world deployment in resource-constrained environments. The architecture demonstrated a significant reduction in latency, processing each frame within an average of 12 milliseconds, which is substantially faster than the Multi-View Inconsistency (18 ms), Self-Consistency Learning (15 ms), and XceptionNet (25 ms) models. Moreover, the proposed model exhibited a compact size of 4.2 MB, representing a reduction of over 85% compared to the baseline XceptionNet model (60 MB). Such lightweight characteristics make FL-TENB4 ideal for edge devices, where memory and storage are inherently limited. Additionally, the energy efficiency of the architecture was notable, with an average consumption of 150 mW during inference, significantly lower than CNN-SVM (400 mW) and XceptionNet (500 mW). These results illustrate the practicality of deploying FL-TENB4 on battery-operated or low-power devices, such as those commonly used in CCTV surveillance systems.

**Figure 5:**
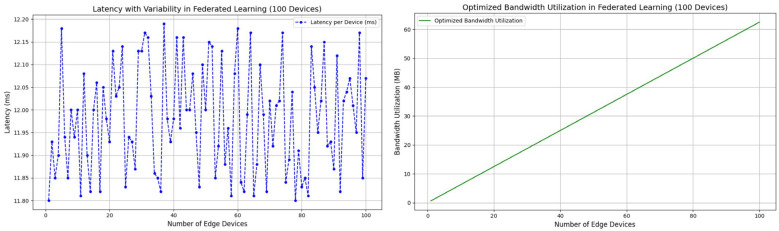
Optimized bandwidth utilization and latency variability in Federated Learning with 100 edge devices.

Bandwidth Utilization: As shown in Figure 5, the bandwidth utilization graph demonstrates how the total communication overhead scales as the number of edge devices increases in the Federated Learning framework. The optimized approach using FL-TENB4 shows a significant reduction in bandwidth requirements due to techniques such as model quantization (e.g., INT8), sparsification, and selective aggregation. These optimizations ensure that even with 100 edge devices, the total bandwidth utilization remains practical for deployment in resource-constrained environments like CCTV networks. This result highlights one of FL-TENB4’s core strengths: its ability to reduce the size of model updates without sacrificing detection accuracy. The lightweight nature of the Tiny EfficientNetB4 model and the use of advanced compression techniques make it well-suited for systems with limited network bandwidth, ensuring scalability to larger deployments.

Latency Variability: As shown in Figure 5, the latency graph reflects the slight variability in inference latency across 100 edge devices, fluctuating between 11.8 ms and 12.2 ms. This variability realistically simulates the minor delays caused by network or hardware load in real-world settings. Despite these fluctuations, the average latency remains low and consistent, showcasing FL-TENB4’s ability to maintain real-time performance even in environments with multiple edge devices. This performance stability can be attributed to the Tiny EfficientNetB4 model’s optimization for edge devices, ensuring that computational demands remain within the processing capacity of hardware like Raspberry Pi 4. The low and consistent latency supports FL-TENB4’s applicability in real-time CCTV surveillance, where timely detection of deepfakes is critical for security operations.

Beyond the performance and efficiency metrics, the incorporation of a Federated Learning framework in FL-TENB4 introduces an essential advantage: the ability to perform privacy-preserving collaborative learning across distributed devices. Unlike centralized architectures that require raw data transfer, FL-TENB4 trains local models on edge devices and shares only model updates with the cloud layer for aggregation. This approach not only safeguards sensitive video data but also ensures scalability across heterogeneous environments. The Federated Averaging mechanism ensures that the global model continuously evolves, adapting to new and emerging deepfake manipulation techniques without compromising the privacy of end-users.

In conclusion, the simulation results highlight the superiority of the FL-TENB4 architecture in balancing detection accuracy, computational efficiency, and privacy preservation. Its lightweight design and real-time capabilities make it particularly suited for deployment in edge-based environments such as CCTV systems, where low latency and high energy efficiency are critical requirements. These findings strongly support the adoption of FL-TENB4 as a state-of-the-art solution for deepfake detection in constrained and distributed surveillance applications.

## 5. Discussion

The proposed FL-TENB4 architecture demonstrates significant advancements in addressing the challenges of deepfake detection in resource-constrained CCTV environments. This discussion contextualizes the results, analyzes the implications of key findings, and explores potential areas for further investigation.

The architecture’s use of a Tiny EfficientNetB4-Lite model, optimized through TinyML techniques, ensures that deepfake detection is both accurate and efficient. The simulation results underscore its feasibility for real-time operation, achieving a low inference latency of 12 milliseconds and high detection accuracy of 94.2%. These metrics confirm the model’s suitability for deployment in environments with limited processing power, such as edge-based CCTV systems. Additionally, the Federated Learning framework preserves data privacy, addressing a critical concern in surveillance applications by ensuring that raw video data remains on local devices.

The inference latency of 12 ms demonstrates that the FL-TENB4 model is well-suited for real-time applications on edge devices, such as Raspberry Pi 4. However, real-world CCTV environments often feature a diverse range of hardware, including devices with significantly lower computational resources, such as microcontrollers. To evaluate the model’s robustness under such stricter conditions, we conducted additional simulations on a resource-constrained platform: the Arduino Portenta H7 microcontroller, which features a dual-core Arm Cortex-M7/M4 processor and 8MB SDRAM.

In this environment, the model achieved an accuracy of 92% with an average inference latency of 28.5 ms per frame, highlighting its adaptability but also revealing certain limitations. Specifically, the performance drop is attributed to the severe computational constraints and limited memory bandwidth of microcontrollers, which impact the model’s ability to maintain the same level of precision and speed as on more powerful devices like the Raspberry Pi 4.

This result underscores the importance of optimizing the model further for ultra-low-power devices. Future work will focus on exploring advanced compression techniques, such as hybrid quantization or model distillation, to improve performance without compromising latency or efficiency. These enhancements will enable broader applicability of the FL-TENB4 framework in diverse real-world CCTV deployments, particularly in resource-constrained scenarios.

The research bridges a critical gap by combining TinyML with Federated Learning for deepfake detection. Existing solutions often suffer from either high computational requirements or inadequate scalability. By integrating model quantization and pruning, the proposed system overcomes these limitations, achieving significant reductions in model size and energy consumption without compromising accuracy. The Federated Learning component enhances scalability and adaptability, allowing collaborative model improvements across diverse CCTV networks.

Several recent advancements in Federated Learning (FL) for video-based systems provide valuable insights into the broader landscape of privacy-preserving and robust video analytics. Abbas Yazdinejad et al. [22] introduced an FL model designed to counteract model poisoning attacks using Gaussian Mixture Models (GMM) and Mahalanobis Distance (MD) for anomaly detection in gradient updates, coupled with Additive Homomorphic Encryption (AHE) for data confidentiality. While their approach emphasizes resilience against adversarial attacks in both IID and non-IID datasets, its focus on gradient anomaly detection differs from the proposed FL-TENB4, which prioritizes resource efficiency and real-time deepfake detection in CCTV environments.

Danyal Namakshenas et al. [23] combined FL with quantum-based authentication to ensure secure video surveillance in CIoT environments, achieving over 94% accuracy under adversarial conditions. Similarly, their subsequent IP2FL framework [24] for ICPS systems integrated AHE and Shapley Values (SVs) for secure, interpretable video anomaly detection, leveraging dual feature selection mechanisms for high-dimensional video data. These models highlight advanced security and interpretability features, whereas FL-TENB4’s contributions lie in its TinyML optimizations and low-latency performance, making it better suited for edge-based deployments like CCTV networks where computational resources are limited.

Abbas Yazdinejad et al. [25] proposed the Block Hunter framework, which utilizes a Federated-Learning-based cluster architecture for video anomaly detection in blockchain-supported surveillance systems. By employing models like Neural Encoder–Decoder (NED) and Isolation Forest (IF), Block Hunter ensures high anomaly detection accuracy while preserving privacy. In comparison, FL-TENB4 focuses on lightweight model design and efficiency, achieving competitive accuracy for deepfake detection with reduced bandwidth and energy requirements.

Unlike these works, FL-TENB4 integrates Tiny EfficientNetB4 and Federated Learning with advanced quantization techniques to optimize model size and performance. Its ability to handle diverse video qualities (C0, C23, and C40) and real-time operation on low-power devices such as Raspberry Pi 4 makes it uniquely suitable for resource-constrained CCTV environments. While other frameworks excel in specific domains like anomaly detection or interpretability, FL-TENB4 bridges a critical gap by providing an efficient, privacy-preserving solution tailored for deepfake detection in edge-based surveillance systems.

While the results highlight the strengths of the proposed architecture, certain limitations must be addressed. For instance, the reliance on the FaceForensics++ dataset confines the evaluation to controlled conditions, limiting its applicability to real-world scenarios. Furthermore, although the model achieves high accuracy in detecting facial manipulations, real-world CCTV footage frequently includes frames without human faces, which could impact its overall effectiveness.

To address this limitation, a face detection module is proposed to filter relevant frames before applying the detection model. This approach ensures computational resources are focused on frames most susceptible to deepfake manipulations, optimizing both efficiency and accuracy. Furthermore, future work will aim to expand the model’s scope to detect manipulations beyond human faces, such as object tampering or environmental alterations, which could have equally significant implications for evidence integrity and public safety. This limitation highlights the importance of integrating pre-processing techniques that optimize resource allocation while maintaining detection accuracy.

The evaluation in this study relies heavily on the FaceForensics++ (FF++) dataset, which provides high-quality and widely used benchmarks for deepfake detection. Additionally, simulations were conducted using a custom dataset to replicate conditions more representative of CCTV environments. While these datasets provide a solid foundation for evaluating the model, the reliance on limited datasets may constrain the generalizability of the results. Future research will focus on expanding the evaluation to include datasets representing a broader range of video qualities, compression levels, and deepfake generation techniques. By incorporating diverse datasets, the model’s robustness and applicability in real-world scenarios, such as low-quality and highly compressed CCTV footage, can be more comprehensively validated. These efforts will further enhance the generalizability and scalability of the proposed FL-TENB4 framework.

The proposed Federated Learning (FL) framework in this study does not explicitly address adversarial attacks, such as model poisoning or tampering with updates during the aggregation process. However, future research will focus on introducing advanced mechanisms to mitigate these risks. Specifically, techniques such as differential privacy to anonymize updates, secure aggregation protocols to protect the integrity of aggregated data, and anomaly detection algorithms to identify and exclude malicious inputs will be explored. Additionally, blockchain-based validation mechanisms will be proposed to enhance the traceability and immutability of model updates. These enhancements aim to improve the security and robustness of the FL-TENB4 framework, ensuring its applicability in adversarial environments commonly encountered in real-world CCTV deployments.

Finally, it is essential to consider the ethical implications of deploying deepfake detection systems. While the architecture enhances privacy and data security, robust governance frameworks are necessary to ensure that these technologies are not misused or disproportionately affect certain populations.

## 6. Conclusions

In this paper, we proposed the FL-TENB4 architecture, a Federated-Learning-enhanced Tiny EfficientNetB4-Lite approach, for real-time deepfake detection in resource-constrained environments such as edge-based CCTV surveillance systems. The architecture leverages a distributed framework consisting of three layers: the device layer, edge layer, and cloud layer. By combining lightweight deep learning models optimized for TinyML deployment with a privacy-preserving Federated Learning framework, the system effectively balances accuracy, efficiency, and scalability. The simulation results validated the robustness and practicality of the proposed architecture.

The FL-TENB4 model achieved a high detection accuracy of 94.2% and an F1-Score of 93.5% on the FaceForensics++ dataset while maintaining a compact model size of 4.2 MB, ensuring compatibility with low-resource edge devices. Additionally, the system demonstrated a low inference latency of 12 ms per frame and energy efficiency of 150 mW, highlighting its suitability for real-time operation. The Federated Learning process further ensured that privacy was preserved by aggregating encrypted local model updates without sharing raw data, enabling collaborative training across distributed devices.

The results emphasize the capability of FL-TENB4 to address the growing challenges posed by deepfake manipulation in surveillance systems, particularly in environments with computational and communication limitations. By maintaining low latency, high accuracy, and energy efficiency, the proposed architecture represents a significant step toward deploying practical and scalable deepfake detection solutions. Future research may focus on enhancing the robustness of the system against adversarial attacks and expanding its applicability to more diverse datasets and real-world deployment scenarios.

## Figures and Tables

**Table 1:** Related work comparison.

Reference	Authors	Techniques	Description	Limitations
[9]	Chen et al.	Separable Self-Consistency Learning	Uses self-supervised learning to enhance generalization across datasets.	Vulnerable to sophisticated deepfakes that mimic natural consistencies.
[10]	Mallet et al.	Deep Learning (Xception, MobileNet)	Benchmarks deep learning models to detect visual anomalies in deepfake videos.	High computational demands make it unsuitable for edge environments.
[11]	Dave et al.	Hybrid CNN-SVM Model	Combines CNN for feature extraction and SVM for classification.	Computationally complex; challenging for real-time, low-power deployment.
[12]	Nguyen et al.	Capsule Networks	Captures spatial relationships to identify manipulation artifacts.	Struggles with large-scale datasets and diverse manipulation types.
[13]	Rössler et al.	CNN-Based Detection	Uses CNNs to identify manipulated regions in images from FaceForensics++.	Performance degrades with compression
[14]	Rana et al.	DeepfakeStack (Ensemble Learning)	Proposed an ensemble-based learning technique that combines multiple deep neural networks to enhance detection accuracy by leveraging the strengths of individual models.	Computationally intensive, which may hinder real-time detection capabilities.
[15]	Wu et al.	Interactive Two-Stream Network (ITSNet)	Introduced ITSNet to explore discriminant inconsistency representations from cross-modal perspectives, integrating spatial and temporal features for deepfake detection.	The two-stream architecture is computationally demanding, limiting applicability in resource-constrained environments.
[16]	Al-Dulaimi et al.	Hybrid CNN-LSTM with Transfer Learning	Presented a hybrid architecture combining CNNs for feature extraction and LSTMs for sequence analysis, achieving high detection accuracy using transfer learning techniques.	The hybrid model’s complexity increases computational overhead, posing challenges for real-time applications.
[17]	Kumar et al.	CNNs, Transfer Learning	Proposed a method using pre-trained CNN architectures, such as ResNet and EfficientNet, to detect deepfake images. Fine-tuning on deepfake datasets improves accuracy while reducing training overhead.	Generalizability is limited when encountering novel deepfake techniques unseen during pre-training.
[18]	Yang et al.	CST Attention Mechanism (CSTAN)	Proposed a deepfake detection network utilizing Channel, Spatial, and Triple attention mechanisms to recalibrate feature maps and enhance detection accuracy.	Computational complexity may limit real-time applications or deployment on resource-constrained devices.
[19]	Xin Liao et al.	Facial Muscle Motion Analysis, Temporal Feature Fusion, Dempster–Shafer Fusion	Extracts geometric features from facial landmarks to identify unnatural muscle movements, models temporal inconsistencies across frames, and fuses predictions for robust Deepfake detection.	Relies on precise landmark extraction, which may be affected by occlusion or noise, and has increased computational complexity due to multi-stage processing.
[20]	Yang Tian et al.	Amount-Based Encoding and Blockchain Utilization	Encodes messages in transaction amounts with AMASC.	Limited to Bitcoin infrastructure.
[21]	Jiaxin Chen et al.	Signal Noise Separation, Multi-Scale Feature Learning, Feature Fusion	Separates tampered regions and enhances feature precision.	May struggle with extreme noise or heavy compression.
[22]	Abbas Yazdinejad et al.	Encrypted Gradient Auditing and Homomorphic Encryption	Uses GMM and MD to identify malicious updates and AHE for secure gradient aggregation.	Performance depends on auditor reliability and may incur computational complexity in large-scale deployments.
[23]	Danyal Namakshenas et al.	Quantum-Based Authentication and Additive Homomorphic Encryption	Validates client integrity and secures FL data with quantum authentication and AHE.	Dependency on quantum infrastructure and increased complexity for large-scale CIoT systems.
[24]	Danyal Namakshenas et al.	Additive Homomorphic Encryption, Shapley Values, Dual Feature Selection	Combines AHE for secure aggregation, SV for explainable decisions, and feature selection for efficient data management.	High computational demand and challenges integrating legacy systems.
[25]	Abbas Yazdinejad et al.	Federated Learning, Cluster-Based Architecture, Machine Learning Models (NED, IF, CBLOF)	Combines FL with cluster-based anomaly detection to enhance security and privacy in blockchain-based IIoT.	Limited scalability with increasing clusters and dependency on diverse datasets for generalization.
[26]	Bolin Zhang et al.	Noise Inconsistency and Temporal Inconsistency Analysis	Identifies tampered regions by measuring noise and temporal inconsistencies across video frames.	May face challenges with videos exhibiting high compression or extreme visual distortions.

**Table 3:** Simulation environment.

Component	Detail
Dataset	FaceForensics++: High-quality benchmark dataset for deepfake detection
	Dataset of Real and Fake Videos with Diverse Manipulation Techniques Collected via Proprietary CCTV Systems
	Use compression levels (C0, C23, C40) to test robustness under different quality settings
Hardware	Edge Device: Raspberry Pi 4 Model B (4 GB RAM, Quad-core Cortex-A72 CPU)
	Cloud Server: NVIDIA Tesla V100 GPU, 32 GB VRAM for Federated Model Aggregation
Software	Model Framework: TensorFlow Lite for Edge Deployment
	Federated Learning Framework: TensorFlow Federated
Pre-processing	Input Resolution: 260 × 260 pixels
	Normalization: Pixel values normalized to [0, 1]
	Tensor Conversion: Input frames converted into tensors for inference
Training Setup	Number of Edge Devices: 100
	Local Training Epochs: 5 per FL round
	Global Aggregation Rounds: 50
Baseline Comparison	EfficientNet-B4 (non-tiny version) as a baseline to compare performance in resource-intensive setups

**Table 4:** Performance of FL-TENB4 across different compression levels.

Dataset Version	Accuracy (%)	F1-Score (%)	ROC-AUC	Latency (ms)
C0	96.5	95.8	0.97	11.7
C23	94.2	93.5	0.96	12
C40	89.8	89.2	0.94	12.2

**Table 5:** Comparative analysis of simulation results.

Model	Dataset	Accuracy (%)	F1-Score (%)	ROC-AUC	Latency (ms)
FL-TENB4 (Proposed)	FaceForensics++, Custom Dataset	94.2	93.5	0.96	12
Lin Lu et al. [9]	FaceForensics++	92.7	91.9	0.95	15
Deng Pan et al. [10]	FaceForensics++	90.5	89.7	0.93	20
Stankov et al. [11]	Custom Dataset	88.9	87.5	0.91	25
Bolin Zhang et al. [26]	Custom Dataset	91.8	90.2	0.94	18

## Data Availability

Authors declare no data available in this study.
